# Are Grid-Like Representations a Component of All Perception and Cognition?

**DOI:** 10.3389/fncir.2022.924016

**Published:** 2022-07-14

**Authors:** Zhe Sage Chen, Xiaohan Zhang, Xiaoyang Long, Sheng-Jia Zhang

**Affiliations:** ^1^Department of Psychiatry, Department of Neuroscience and Physiology, Neuroscience Institute, New York University School of Medicine, New York, NY, United States; ^2^Department of Neurosurgery, Xinqiao Hospital, Army Medical University, Chongqing, China

**Keywords:** grid cell, cognition, perception, attractor, recurrent neural network

## Abstract

Grid cells or grid-like responses have been reported in the rodent, bat and human brains during various spatial and non-spatial tasks. However, the functions of grid-like representations beyond the classical hippocampal formation remain elusive. Based on accumulating evidence from recent rodent recordings and human fMRI data, we make speculative accounts regarding the mechanisms and functional significance of the sensory cortical grid cells and further make theory-driven predictions. We argue and reason the rationale why grid responses may be universal in the brain for a wide range of perceptual and cognitive tasks that involve locomotion and mental navigation. Computational modeling may provide an alternative and complementary means to investigate the grid code or grid-like map. We hope that the new discussion will lead to experimentally testable hypotheses and drive future experimental data collection.

## Introduction

Our brains constantly build spatial representations of objects surrounding us in our daily lives, and enable us to see, touch and navigate effortlessly. These neural representations that are often referred to as cognitive maps play critical roles in perception, memory and planning. Neuroscientists have first identified that the hippocampal-entorhinal network of freely foraging rats and bats forms a cognitive map in either 2D or 3D environments (O'Keefe and Dostrovsky, 1971; Taube et al., 1990; Hafting et al., 2005; Yartsev et al., 2011; Rowland et al., 2016; Ginosar et al., 2021; Grieves et al., 2021). The entorhinal cortex (EC) provides the primary cortical input to the hippocampus (Van Strien et al., 2009). In spatial navigation, grid cells in the EC are believed to generate a path integration input to hippocampal place cells (Fuhs and Touretzky, 2006; McNaughton et al., 2006; Burgess et al., 2007; Burgess, 2008; Burak and Fiete, 2009). Specifically, grid-like firing patterns provide a mechanism for dynamic computation of self-position based on continuously updated information about position and direction.

**Figure 1:**
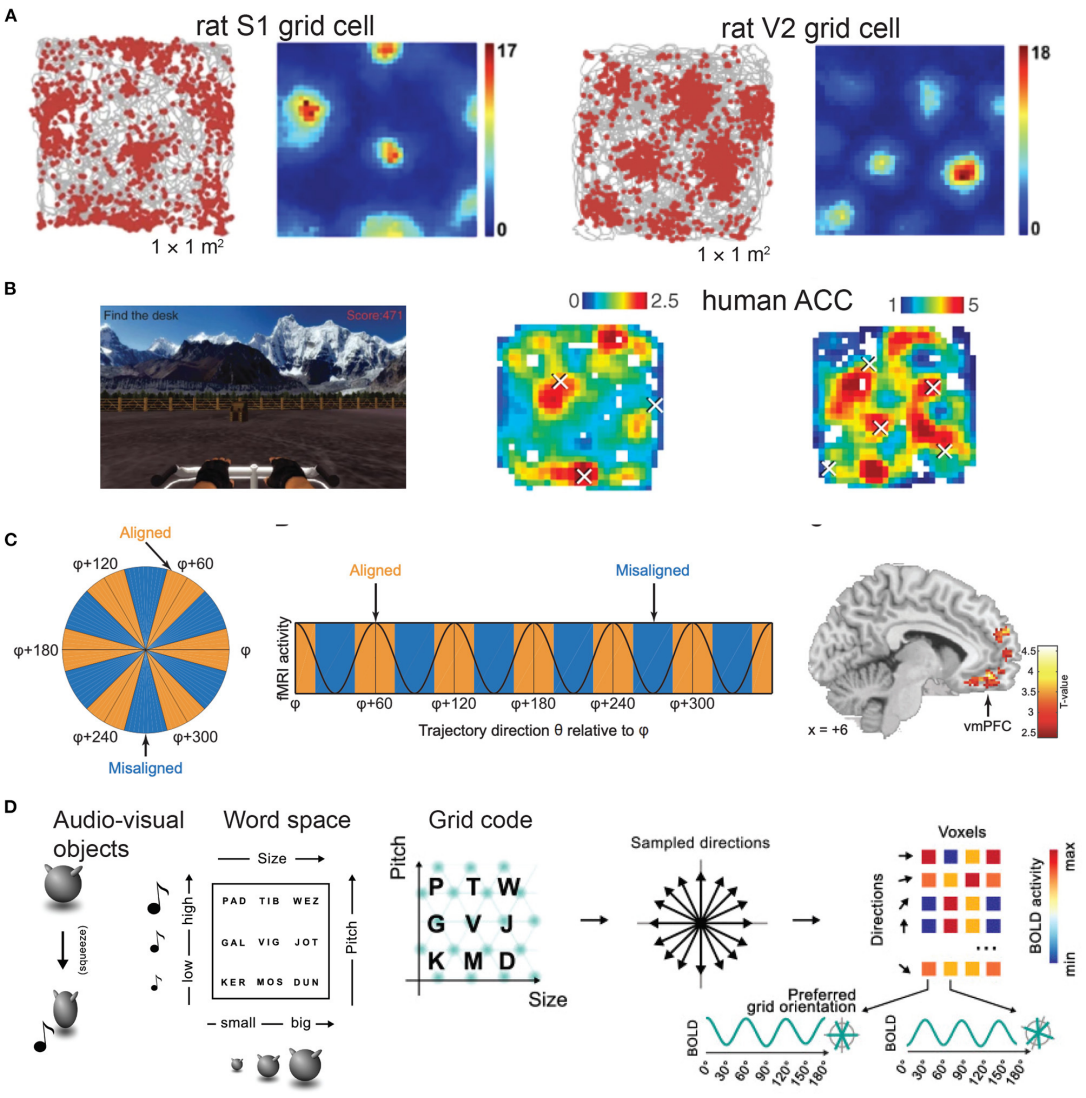
Grid-like responses across rodent and human brains. **(A)** Electrophysiological data show grid-like firing patterns from the rat primary somatosensory cortex (S1) (images are modified from Long and Zhang, 2021, *Cell Research*; reprinted with permission, Creative Commons CC BY license) and the secondary visual cortex (V2) while animals navigated in an open field arena. Color bar shows the firing rate in spikes/s (figure is modified from Long et al., 2021a, bioRxiv). **(B)** Human invasive electrophysiological data show grid-like representations in the anterior cingulate cortex during a virtual navigation task. Color bar shows the firing rate (Hz) (figures are modified from Jacobs et al., 2013, *Nature Neuroscience*; reprinted with permission, from the authors and Springer Nature). **(C)** Human fMRI data show grid-like representations in the ventromedial prefrontal cortex (vmPFC) during a two-dimensional olfactory navigation task (figures are modified from Bao et al., 2019, *Neuron*; reprinted with permission, from the authors and Elsevier). **(D)** Left panel: example of audiovisual object—Nine audiovisual objects were created by manipulating the size of a shape and the pitch of an associated sound, produced during a short squeezing animation. Middle panel: Each audiovisual object was given an abstract name, that could be conceived as a location in a 2D word space. Right panel: Illustration of detection of grid code (figures are modified from Vigano et al., 2021, *Neuroimage*; reprinted with permission, from the authors and Elsevier).

Allocentric (world-centered and viewpoint-invariant) and egocentric (self-centered) representations of space define two distinct reference frames and coordinate systems for coding environmental features (Bicanski and Burgess, 2020; Wang et al., 2020). Our brains dynamically integrate allocentric information and employ memory-guided movements. One noteworthy structure of these neural representations is the discovery of grid-like firing patterns in single neurons from the medial entorhinal cortex (mEC) in mice, rats and bats during freely foraging (Hafting et al., 2005; Fyhn et al., 2007, 2008; Yartsev et al., 2011; Ginosar et al., 2021; Grieves et al., 2021) (Figure 1A), from the pre- and parasubiculum of rats (Boccara et al., 2010), from the mEC and cingulate cortex in human patients during virtual reality exploration (Jacobs et al., 2013; Nadasdy et al., 2017) (Figure 1B), as well as the grid-like responses of fMRI BOLD signals in human neuroimaging during cognitive tasks and mental stimulation (Doeller et al., 2010; Constantinescu et al., 2016; Horner et al., 2016; Bellmund et al., 2018a; Nau et al., 2018; Bao et al., 2019; Kim and Maguire, 2019) (Figure 1C). Notably, grid-like representations not only appear in the human mEC, but also in other traditionally thought non-spatial frontal brain areas, such as the human orbitofrontal cortex (OFC), ventromedial prefrontal cortex (vmPFC) and anterior and posterior cingulate cortex (ACC and PCC) (Constantinescu et al., 2016; Bao et al., 2019). Recently, grid cells have also been discovered in the rat primary somatosensory cortex (S1) (Long and Zhang, 2021) and the secondary visual cortex (V2) (Long et al., 2021a). What are the roles and functional significance of these sensory cortical grid cells? In this paper, we make a few speculative accounts hoping to inspire outside-of-the-box thinking and stimulate cross-disciplinary discussions among the neuroscience community. Specifically, we will provide both microscopic and macroscopic system views of grid-like responses, which match the rodent electrophysiology and human fMRI recordings reported to date. We will focus our discussion on the sensory component given the predominant animal literature, and further extend the discussion to the abstract conceptual domain in cognition. In this short opinion article, we make no attempt to review all experimental findings or all computational models of grid cells, but pay specific attention to generalized grid codes beyond the hippocampal-entorhinal system and further argue their universal roles in perception and cognition. In addition, we outline a few theory-driven computational mechanisms (such as predictive representations and attractors emerged from recurrent computation) that may explain the rationale of generating grid-like maps.

## Common Principles and Spatial Mapping in Sensory Perception

We notice that there are several common principles shared by various types of sensory perception. First, spatial localization is a common theme in sensory perception. We live in a three-dimensional world. Nearly all aspects of visual, auditory, touch and olfactory perception, involve “what” and “where” processing that requires hippocampal localization of an object in space (Schlag and Schlag-Rey, 2002; Bizley and Cohen, 2013; Brooks and Medina, 2017; Poo et al., 2022). For instance, when we reach and search for an object in the dark, our brains are aimed to build a representation map of the object (or the parts of the object) relative to the reference frame. Therefore, it is not impossible that the somatosensory cortex will respond in grid-like patterns when different parts of the objects are touched, so that all combined neural responses form a coherent allocentric representation of the object. Extending the original idea of cognitive map for spatial landscape (Tolman, 1948), the non-spatial information can be organized among visual, olfactory, social and imaging concepts (Bellmund et al., 2018b; Herweg and Kahana, 2018; Raithel and Gottfried, 2021).

Second, allocentric and egocentric representations have been found in many sensory cortices. To date, multiple lines of evidence have suggested that the brain may use grid-like representations for allocentric perception. Human fMRI experiments have reported that the somatosensory cortices are activated when subjects watched video clips of a hand being touched in egocentric as well as in allocentric perspectives, suggesting that somatosensory responses differ depending on the perspective of the observed touch (Schaefer et al., 2009). In the case of visual perception, although a visual input is primarily egocentric and the primary visual cortex (V1) is viewed as an egocentric cognitive map (Linton, 2021), some other forms of visual perceptions are allocentric and independent of the observer's vantage point or motion (Wexler, 2003). Additional human studies have shown that vision is necessary for allocentric spatial coding during development for visually impaired children (Martolini et al., 2020). Interestingly, V2 is strongly activated during allocentric reach tasks (remembering the target location relative to a visual landmark) in human fMRI experiments, whereas the parieto-frontal cortex is activated during egocentric reach tasks (remembering the absolute target location), suggesting that the location of a remembered reach target can be encoded in both allocentric and egocentric reference frames (Chen et al., 2014a). Likewise, egocentric and allocentric representations have been found in the auditory cortex of freely moving ferrets (Town et al., 2017). The auditory cortex can implement sound localization relative to the head and the world. Upon hearing a sound in a room, we can describe its location relative to ourselves (e.g., “the sound comes from my right side”) or relative to the room (e.g., “the sound source is close to the eastern window”). In the recent studies of the S1 and V2 of freely forging rats (Long and Zhang, 2021; Long et al., 2021a), we have found conjunctive coding of head direction in grid cells, suggesting that sensory cortical neurons have mixed selectivity in spatial representations; additionally, these grid-like responses are not disrupted from the absence of vibrissae or visual input.

Third, the emergence of cognitive maps in sensory cortices may be driven by an iterative learning process *via* a perception-action loop. Notably, all sensory perception is dynamic (constantly in motion) and engage in sensorimotor coordination or integration. The perception-action cycle is rooted in all goal-directed behaviors, with a circular information flow that links an organism to its environment. During this dynamic process, a series of cognitive models for sensory perception, attention, memory and sensorimotor integration are executed to perform task behaviors. A fundamental building block of the cognitive map is the grid-like representation of objects in a high-dimensional multi-sensory space. In terms of the computational mechanism, emergent grid-like representations may arise from reinforcement learning (Stachenfeld et al., 2017), or from training recurrent neural networks (RNNs) on navigation or multiple normative tasks from supervised learning (Banino et al., 2018; Cueva and Wei, 2018; Sorscher et al., 2020; Zhang et al., 2022). Specifically, the grid-like representation is the eigenvector of the state-space transition matrix derived by the successor representation (SR) algorithm, and it is explained as a low-dimension sparse representation of the cognitive map. Whereas in RNNs, recurrent dynamics can generate stable ring or torus-like attractors that are associated with the grid patterns (Sorscher et al., 2020; Zhang et al., 2022). Although the existing computational theories of grid cells have focused on spatial navigation and path integration, conceptual analogies can be made between navigating in a Cartesian space and navigating in other physical or non-physical spaces. The movement in the latter case is associated with hand movement (in touch perception), head movement (in auditory perception) or both (in visual perception). Grid-like responses may also have conjunctive representations in the brain, as recent studies have shown that mEC grid cells in mice or rats can encode behaviorally relevant information, such as the generic continuous task-variables (sound frequencies), reward and goal locality (Aronov et al., 2017; Boccara et al., 2019; Butler et al., 2019). This “active perception” notion is in line with the universal perception-action loop inherent in all goal-driven behaviors.

## From Perception to Cognition

Perception and cognition play different roles in mental processes. While perception emphasizes sensing information around the environment through organization and identification, cognition involves attention, memory, reasoning and knowledge representation. While these two processes are interleaved, perception consists of more bottom-up effects, whereas cognition involves more top-down processing. In the literature, the notion of *generalized cognitive maps* has been proposed for knowledge representation and concept learning (Behrens et al., 2018; Mok and Love, 2019; Dang et al., 2021). Grid-like responses have been found in the human brain beyond the traditional navigation task (Constantinescu et al., 2016; Vigano et al., 2021). It is natural to envisage generalized versions of cognitive maps that organize conceptual knowledge as the analogs of world-centered representations of the environment.

Take vision as an example, recognition of a set of image or video sequences can be viewed as navigation in a low-dimensional feature space, where the “distance” between points in the feature space characterizes the similarity of the high-dimensional visual stimuli (Zhang et al., 2022). Visual memory consists of holding visual images and spatial perception in the feature space (“fixed points”). Reasoning or planning, on the other hand, introduces an additional level of dynamic thought processes conditional on the stimuli and other task variables. Specifically, both spatial and non-spatial relational inference can be cast as structural generalization in the *Tolman-Eichenbaum machine* (TEM) (Whittington et al., 2020), the transition probability between the observed or latent states characterizes the dynamics of spatial or mental sequences (Chen et al., 2014b; Kurth-Nelson et al., 2016; Nour et al., 2021).

In a recent fMRI experiment where subjects were instructed to compare newly learned words that were referring to audiovisual object, grid-like and distance code were found in the mEC and the PFC/OFC/cingulate cortex, respectively. Specifically, the grid code represents the relative angular positions of words in the “word space” (Figure 1D). Therefore, the abstract concepts are conceivable as points of an internal map (where distance represents similarity)—which, similar to the physical space, can be mentally navigated (Dang et al., 2021; Vigano et al., 2021). In another fMRI experiment, it has been found that humans use a grid-like code (hexagonal modulation) in the entorhinal cortex and dorsal mPFC to perform discrete decisions in the reconstructed abstract space (Park et al., 2021). This line of work has generalized the concepts from behaviorally relevant, continuous, non-spatial stimulus dimensions (such as the sound frequency, odor concentration, car size and engine power in a conceptual “car space” (Aronov et al., 2017; Bellmund et al., 2018a; Bao et al., 2019) to abstract and discrete problems. In the case of Park's experiment (Park et al., 2021), the trajectories for novel inferences corresponded to a 2D cognitive map of social hierarchy.

One of the new (and old) theories for generalized cognitive maps speculates that the brain employs grid cell-like mechanisms to navigate in an abstract “concept” space and learn the structure of the world (objects), and the abstract concepts are represented *via* reference frames that are implemented by cortical columns (Mountcastle, 1978; Hawkins et al., 2019). In analogy to spatial navigation, the reference frame is a map that enables the brain not only to see, to touch, and to hear effortlessly, but also to make timely sensory-motor predictions. In vision, therefore, grid-like computation can be implemented in visual cortical columns to track the location of visual features relative to the objects being viewed. Similarly, grid-like computation can be implemented in the somatosensory cortical columns to track the location of tactile features relative to the objects being touched. In the case of rat V2 grid cells, theory-driven hypotheses have been confirmed by preliminary experimental findings (Hawkins et al., 2019; Long et al., 2021a). In the previous report, S1 grid cells were recorded across Layer IV-VI of the rat S1HL (hindlimb) area, and V2 grid cells were recorded across superficial and deep layers of the rat V2M area (Long and Zhang, 2021; Long et al., 2021a); and some evidence has shown clustered or columnar structures in the primary and secondary areas of these sensory cortices (Horton and Adams, 2005; Laramee et al., 2013; Hubatz et al., 2020). However, it remains to be determined how these sensory cortical grid cells are generated and adapted during the course of learning. Meanwhile, action and thinking provides an abstract form of movement interacting with the external world to enable closed-loop adaptation. Demystification of the brain intelligence theory can further motivate the development of artificial intelligence and machine learning. In fact, the theories of minicolumn and coordinate frame are not completely unfamiliar. Hinton's “Capsule Network” theory was also built upon the notion of the “coordinate frame” in computer vision. In this theory, minicolumns would enable computers to represent and detect multidimensional features of objects, performing coincident voting and view-invariant recognition (Hinton, 2021). Future developments of theories and biologically realistic computational models that implement grid-like location-based computations across all sensory cortices or higher-order cortices would provide deeper insight into the mechanism (Shilnikov and Maurer, 2016; Cueva and Wei, 2018; Yu et al., 2021; Zhang et al., 2022).

## Mysteries of Grid Cells in Sensory and Frontal Cortices

Several fundamental questions remain for grid cells in the cerebral cortex. First, does the grid phenomenon serve as a general solution to “spatial localization” problems of all perception and cognition across higher-order cortical areas as well as sensory cortical areas? Furthermore, does that arise from evolution across species (e.g., bats, rats, monkeys, and humans) or emerge from general-purpose experience-dependent learning? One theory has suggested that the mEC may operate as a generalist circuit that obey computational design principles resembling those governing other higher cortical areas (Hardcastle et al., 2017).

Second, is there a computational unit that implements regular periodic grid firing ubiquitous in the cortex; if so, what are the symmetry-breaking mechanisms and prerequisites? One theory postulated that grid cell behaviors are underlay by the self-organized two-dimensional synaptic matrix with periodic boundary conditions; accordingly, grid-like patterns of neural activity might be present in the immature cortex during early prenatal development, and that these activity patterns guide the development of the periodic synaptic matrix through a competitive synaptic plasticity mechanism (McNaughton et al., 2006). Recent theories based on RNNs based on supervised learning also suggested alternative computational mechanisms (Cueva and Wei, 2018; Sorscher et al., 2020). Excitation-inhibition (E/I) balance and Dale's principle in synaptic connections may also provide additional biological constraints to understand the recurrent circuits for grid codes (Zhang et al., 2022).

Third, what are the natural metrics for general-purpose grid computation in the brain? The brain is capable of integrating self-motion cues derived from locomotion, vestibular activation and optical flow (path integration) for the purpose of spatial learning. Although space and time are commonly understood as Newtonian concepts, these observer metrics may be distorted when applied to the brain. Various human experiences have confirmed that our internal perception of space and time (similarly for speed and direction) can vary according to specific conditions (Wittmann, 2009; Buzsaki and Llinas, 2017).

While noticing there are still limited experimental grid cell data in sensory and frontal cortices, here we outline the plausible mechanistic and computational principles underneath the grid-like computation in the sensory and higher-order cortices. We will first discuss the known facts in the EC (belonging to the so-called “old cortex”), which is the main interface between the hippocampus and neocortex. The EC consists of layered architectures, with each layer receiving from and projecting to differential targets (Witter et al., 2017), and the majority of grid cells in rodents are observed in the layer II of mEC. In the mEC, a hierarchy of discrete “grid cell modules” has been discovered to be distributed across the longitudinal axis with multiple grid scales (Barry et al., 2007; Stensola et al., 2012; Naumann et al., 2018), and the path integration may enable animals to self-localize even in the darkness. Such a modular structure can emerge from an attractor mechanism through dynamic self-organization (Kang and Balasubramanian, 2019). The mEC grid cells of head-fixed monkeys can also encode space during visual exploration without locomotion in a free-viewing visual memory task (Killian et al., 2012). Additionally, the structure of grid cell firing supports a learned topology of ordered experience rather than a rigid coordinate frame that is bound to measurements of the physical world (Rueckemann et al., 2021). In virtual reality experiments of mice, visual inputs and physical motion inputs could be dissociated (Chen et al., 2019): the mouse mEC grid cells mostly reflect a greater influence of physical motion, while mouse hippocampal place cell firing patterns predominantly reflect visual inputs.

In rodent experiments, recent preliminary data have found that grid cells in the rat V2 and S1 respond to self-location in space in a similar manner to the hippocampal-entorhinal system (Long and Zhang, 2021; Long et al., 2021a,b). However, this finding did not exclude the possibility that all or part of these V2 and S1 grid cells also respond to visual or tactile scenes. As a matter of fact, the presence of category-selective cells and multisensory cells has been widely reported in sensory cortices (Roy, 2017). It remains unknown whether the mutually orthogonal V2 or S1 grid cells in physical space (in either spatial frequency or phase) also preserve the firing orthogonality in the visual or somatosensory feature space. Future control experiments that record these sensory cortical grid cells under different experimental stimuli would be able to demystify the puzzle. Not only S1 and V2 place cells and head-direction cells have been found in freely foraging rats (Long and Zhang, 2021; Long et al., 2021a, 2022), the spatial modulation of place cells and grid cells in the rat S1 and V2 can persist in the absence of sensory input (e.g., whisker trimming and darkness); these results suggest the independence and robustness of these spatially-modulated neurons in sensory cortices. Furthermore, theta oscillations have been found in both rat S1 and V2 areas, providing a source of speed and acceleration inputs. Head-direction signals reported in the rat S1 and V2 may receive indirect directional input from the RSC and postsubiculum (Taube, 2007), or may be derived from the sensorimotor input, such as the visual optical flow (Zhang et al., 2022). Additionally, the visual cortex also provides an important source of self-motion information to mEC grid cells; such multimodal signals may play a vital role in spatial perception (Campbell and Giocomo, 2018). Grid cell coding in the limb/shoulder areas of the rodent S1 might reflect either locomotion feedback from motor areas or ascending proprioceptive signals used for path integration in S1; in other words, these grid signals might be still dependent on sensory inputs derived from locomotion. However, a complete mechanistic dissection of S1 and V2 grid cells in rodents would require causal manipulation of their downstream or upstream structures, including the sensory thalamus, V1 and S2, and possibly the primary and secondary motor cortices (M1 and M2). Equally important, it would be good to check whether grid cells are present in the sensory cortices of freely flying bats. In addition to the traditional hippocampal-entorhinal system, many cortical-subcortical structures in the limbic circuits are involved in spatial memory and navigation, including the OFC, piriform cortex, and anterior thalamic nuclei (ATN) (O'Mara and Aggleton, 2019). Specifically, neurons in the rat OFC can form spatial representations of future goal destination in conjunction of location-selective tuning (as high as 80%) (Basu et al., 2021). Neurons in the rat piriform cortex have mixed tunings and can carry spatial representation of a learned cognitive map, in addition to the odor identity (Poo et al., 2022). To date in addition to V1 (Flossmann and Rochefort, 2021), it remains unclear whether the descending pathways from limbic navigation circuits indirectly influence the spatial tuning in sensory cortices of rodents (S1 and V2).

What are those cortical grid cells needed in spatial navigation? What is the functional significance of these cortical grid cells (especially given their small proportions)? We speculate that the sensory cortical grid cells emerge from a generalized computation principle similar to path integration. Take vision as an example, the visual cortex of freely foraging animals may constantly integrate speed and direction information from dynamic visual scenes (e.g., through computation of visual optical flow); this information can be used to update self-location. We speculate that these sensory cortical grid cells are complementary yet functionally independent from the mEC grid cells. Multi-site electrophysiological recordings in the future rodent experiments may be able to test this hypothesis.

Despite many unknowns, it is not unreasonable to envision that similar functional modules are distributed across sensory cortices. One proposed theory is that the sensory cortical columns consist of neurons that perform functions similar to grid cells, which will activate according to the location of the column's input relative to the external reference frame (either physical or abstract location) (Roy, 2017; Hawkins et al., 2019). These columnar structures can be modeled by a densely intra-connected subnetwork of excitatory neurons in computer simulations (Zhang et al., 2022). The sensory input is likely to be multisensory (e.g., visuospatial or audiospatial). In the case of non-spatial or abstract input, the attribute space will replace the traditional physical space to define the cognitive maps for navigation in the abstract feature space. At the cellular level of animal studies, the mixed selectivity and/or multiplex information coding has been reported not only in the visual and somatosensory cortices (Goris et al., 2015; Kim et al., 2019; Lankarany et al., 2019), but also in the PFC, OFC and ACC (Hayden and Platt, 2010; Rigotti et al., 2013; Fusi et al., 2016; Hirokawa et al., 2019). However, a systematic search of grid-like responses across sensory and high-order cortices and characterization of their conjunctive representations remains to be completed. Given their multiple roles in sensory processing, it is likely that a subset of sensory cortical neurons is recruited to perform grid-like computation in representing the reference frame, but their tuning properties are dynamic and depends on the task context (“dynamic resource allocation” hypothesis).

At the macroscopic level, higher-order cortical grid responses have only been identified in human fMRI experiments. It is mindful to remind that all designed human task behaviors navigating in the abstract feature space involve very few feature attributes. Therefore, it remains unknown how this finding generalizes to a less-constrained cognitive task setting. The very possibility is that the lack of structure in tasks brings a difficulty of detecting generalized grid codes. Moreover, whether such grid firing patterns can be rediscovered by rodent electrophysiology remains unconfirmed. Conceptually, higher-order cortices can use the location-based framework to represent and organize knowledge and social hierarchy (Park et al., 2021). Similar to hippocampal sparse encoding of spatial memories, sparse representations of higher-order cortical population activity may encode abstract behavioral concepts or support flexible cognition and behavior (Constantinescu et al., 2016; Bellmund et al., 2018b; Raithel and Gottfried, 2021). However, it is also worth pointing out that the fMRI-BOLD signal does not directly measure the neuronal activity, and therefore it cannot be interpreted in the same way as the electrophysiological signal (i.e., spiking activity). Therefore, spiking data from human participants (such as epileptic patients) would provide more confirmation for the grid responses. To date, direct evidence of single-unit grid representations from the mEC or any other structures in conceptual spaces has not yet been discovered, partially due to the limited accessibility of human brains in clinical settings.

**Figure 2:**
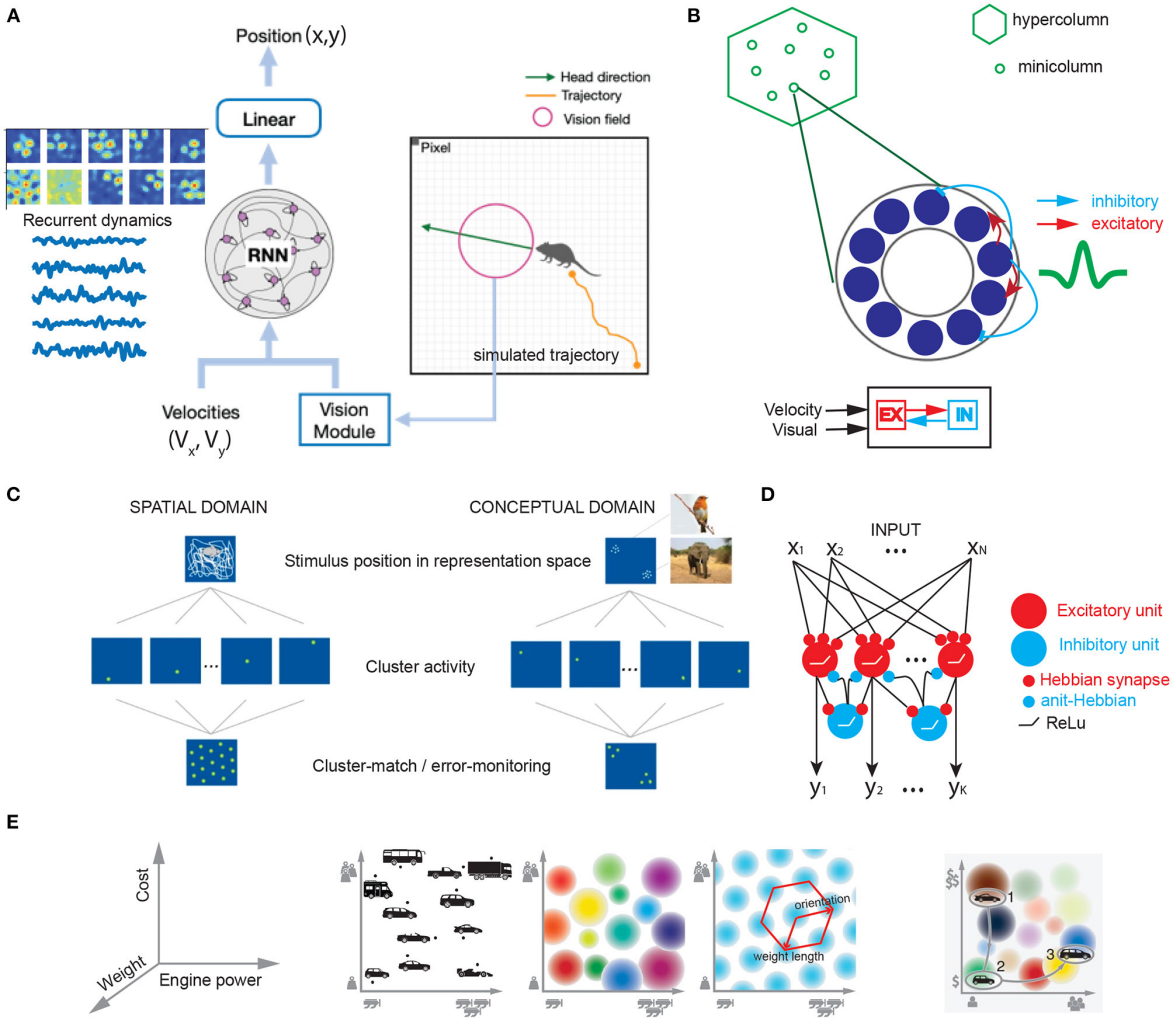
Computational models that explain grid-like computation in spatial and conceptual domains. **(A)** To model the grid cells in the rat V2 visual cortex, we trained an excitatory-inhibitory (E/I) recurrent neural network (RNN) using both velocity input (V_x_, V_y_) and visual input of varying dimension (based on dimensionality reduction from PCA) to decode a simulated agent's trajectory (x,y) in an open field environment. Emergent grid-like responses were found in the RNN's hidden units (Z.S. Chen, Data unpublished). **(B)** A schematic of continuous attractor model for V2 grid cells based on excitatory-inhibitory neuron population interaction. **(C)** Schematic of clustering in spatial and conceptual domains based on the cluster-monitoring/error-monitoring mechanism (figures are modified from Mok and Love, 2019, *Nature Communications*; reprinted with permission, from the authors and Springer Nature). **(D)** E/I feedforward neural network for clustering or learning similarity-preserving map based on local Hebbian rules (Sengupta et al., 2018). **(E)** Illustration of grid cells in cognitive space. Left: 3D feature space that defines independent dimensions satisfying geometric constraints for vehicle. Middle left: 2D space spanned by the dimensions of engine power and car weight. Middle center: Multiple place cells with different firing fields. Middle right: single grid cell with regular periodic firing field. Right: Navigation in a continuous cognitive “car” space (figures are modified from Bellmund et al., 2018a, *Science*; reprinted with permission, from AAAS).

To investigate why and how grid cells emerge in sensory cortices or higher-order cortices, combining theory-driven and experimental investigations can help provide new insight into such inquiry. Complementary to experimental investigations in animals and humans, computational modeling provides a valuable approach to understand the computational mechanism of grid cells. Based on the SR theory, grid representations has been suggested for planning and higher-level cognition, which serve as the basis for learning and representing the experienced relationship between entities (Yu et al., 2021). The eigenvectors of SR can facilitate generalization in novel contexts (“transfer learning”) and represent a factorized task structure in the cognitive map. Recent work has shown that grid cells may emerge from the hidden units of trained RNNs that predict 2D position based on the velocity input (Banino et al., 2018; Cueva and Wei, 2018). Along the same line, a new TEM-driven deep neural network architecture has been developed to model place and grid codes (Whittington et al., 2022). This directly support recurrent attractor dynamics and path integrator in the cognitive map. Recently, our own investigations have also shown that the emergent grid-like responses are preserved by augmenting additional visual input to the RNN (Figure 2A), where the grid-like responses are robust to the visual input and speed representation; additionally, the imposed network connectivity topology and sparsity onto the computational model can change the representation of grid codes and attractor states (Zhang et al., 2022). Recent data from the mouse mEC have demonstrated that sensory inputs rather than visual inputs can support grid cell firing even in complete darkness (Dannenberg et al., 2020). Furthermore, it is possible to conduct computer simulations on other non-spatial tasks and explicitly test the sufficient and necessary conditions of grid cell representations. Grid codes may be able to generalize experiences and make appropriate decisions in novel conditions to accommodate behavioral flexibility (Yu et al., 2021). It is hoped that various continuous attractor models for mEC grid cells previously proposed in the literature (e.g., Figure 2B) can be adapted to accommodate the new task setting or assumptions. There is an alternative approach to modeling macroscopic “grid fields” by generalizing single neurons to neural ensembles from the same module with similar orientation and phase (Rosay et al., 2019). Additionally, self-organized domain-general learning algorithms that explain the emergence of grid cells in both spatial and conceptual domains are appealing for sensory and high-order cortices (Figure 2C; Mok and Love, 2019). Similarity matching or learning may be some universal yet biologically plausible principles implemented in the brain (Figure 2D; Sengupta et al., 2018; Pehlevan and Chklovskii, 2019), and grid representations may provide efficient similarity search strategies. In the conceptual domain, cognitive space is defined by independent dimensions that define geometric constraints of the object (e.g., car, Figure 2E); navigation in a continuous cognitive space will trigger the activation of “place cells” and “grid cells”. Ultimately, linking computational models with experimental data with biological constraints will be the goal of future modeling effort.

## Prediction

**Figure 3:**
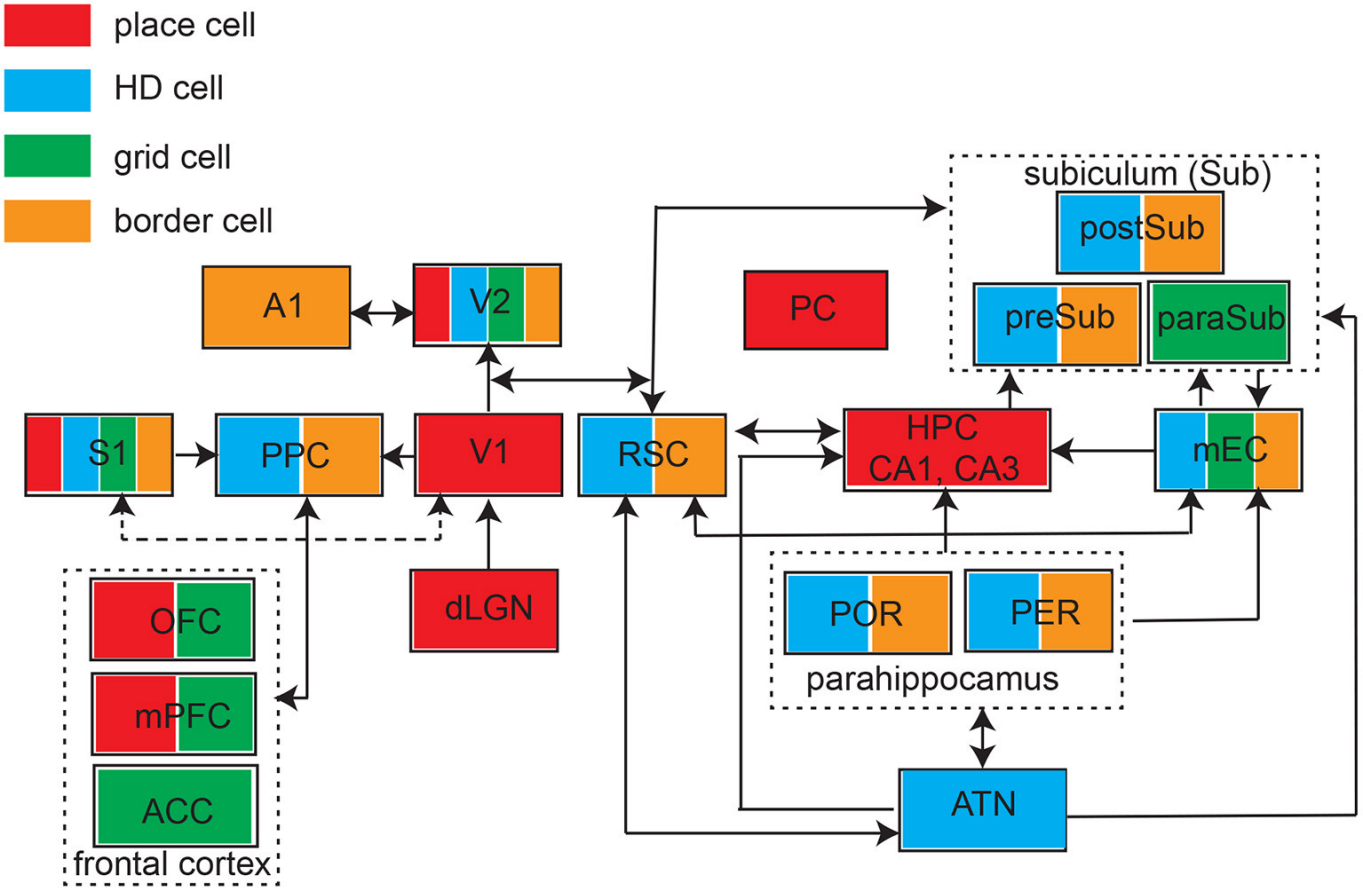
Schematic of identified brain structures with four major types of spatial tunings. ATN, anterior thalamic nuclei; S1, primary somatosensory cortex; PPC, posterior parietal cortex; V1, primary visual cortex; V2, secondary visual cortex; A1, primary auditory cortex; PC, piriform cortex; RSC, retrosplenial cortex; HPC, hippocampus; mEC, medial entorhinal cortex; POR, postrhinal cortex; PER, perirhinal cortex; preSub, presubciculum), paraSub (parasubiculum); postSub, postsubiculum; OFC, orbitofrontal cortex; mPFC, medial prefrontal cortex; ACC, anterior cingulate cortex. Arrow indicates the connectivity.

The past few years have witnessed growing experimental evidence of spatial modulated responses across many brain areas (Figure 3). However, identification of place-like or grid-like patterns in the brain can be relatively arbitrary threshold phenomena, it might not be completely surprising to find such patterns in the brain if proper detection methods are used. A traditional method for detecting grid responses are based on spatial autocorrelation, but this method may generate false positives. A careful control study by random spike or field shuffling (Barry and Burgess, 2007), such as randomizing the temporal structure of spiking while preserving the rate, may help reduce the false detection rate using a strict detection threshold. On the other hand, one important reason for missing true positives is that researchers did not explicitly look for “grid responses” in the non-traditional brain regions; another possible reason is that the limitation of experimental design in many sensory or cognitive tasks (e.g., head-fixed experiments). Therefore, without careful experimental designs, grid patterns can be missed because of the mismatched grid scale of firing patterns with respect to the environmental enclosure (Stensola et al., 2012). Unlike mEC grid cells, sensory cortical grid cells show more diverse and heterogenous responses, and seem to be sparsely distributed (i.e., not densely distributed in a specific cortical layer). Increasing unit yields by high-density probes or large-scale imaging in rodent studies may potentially enhance the opportunity to identify the ensembles of grid cells in sensory cortices (Gardner et al., 2022; Obenhaus et al., 2022; Zong et al., 2022). In human fMRI studies, developing rigorous analyses for detection of grid-like coding and understanding conceptual spaces would be crucial to advance this research area (Kriegeskorte and Storrs, 2016).

Given many knowns and unknowns discussed in the paper, we would like to make several experimentally testable predictions based on the published data available to date. These speculative hypotheses, once being rigorously tested, will improve current understanding of “generalized cognitive maps” for perception and cognition.

### Prediction 1

*Grid-like firing patterns will be discovered in the auditory cortex*. It has been known that the hippocampal-entorhinal neurons in mice can encode non-spatial task variables in the tone space (Aronov et al., 2017). It is also well known that bats and rodents use echolocations to help spatial navigation in the dark. A natural question is that whether similar auditory grid cells exist as in S1 and V2 grid cells in bats or rodents. The auditory cortex has direct projections from V2 and S1 and shares cross-modal responses. Prior studies have shown that spatially selective neurons in the auditory cortex and the midbrain superior colliculus (SC) of bats can form 3D representations of space (Greiter and Firzlaff, 2017; Kothari et al., 2018). It is also possible to find subset of auditory cortical neurons have conjunctive firing properties for representations (maps) of physical and sound features (“tonotopic map”). This may be experimentally tested in freely foraging rats or bats (*via* large-scale electrophysiology or calcium imaging), where the location of space can be paired with distinct sound frequencies. However, the design of human fMRI testing may be more challenging based on virtual reality experiments.

### Prediction 2

*Grid-like responses will be found in the human somatosensory and visual cortices*. Similar to the animal's experiment, this prediction may be tested in human fMRI experiments in virtual reality settings. First, it is worth reexamining the previously collected fMRI data beyond the mEC region. However, due to the mixed selectivity of somatosensory and visual neurons, detection of grid-like responses in somatosensory and visual cortices in fMRI experiments may prove difficult. Another consideration is the design of control experiments to decouple sensory and spatial components (similar to the darkness control experiment in rats). Therefore, new experimental design will be the key of future investigations.

### Prediction 3

*Grid cells will be found in the frontal cortex*. If the frontal cortex is engaged in representing knowledge in a generalized cognitive map, it is not completely impossible that animals use a similar principle as shown in human fMRI findings. This prediction may be tested in freely foraging rodents and monkeys, as animals have demonstrated the capability of learning the category knowledge (Fize et al., 2011; Goltstein et al., 2021); but it remains unknown what kind of abstract knowledge (which is often represented by single or multimodal sensory stimuli) is most effective for specific species. In the case of monkey electrophysiological recordings, large unit yields may also prove crucial for the discovery of grid cells because of possibly sparse grid-cell representations.

### Prediction 4

*There may be universal functions across sensory cortices that implement egocentric-to-allocentric transformation*. In light of our discussion of allocentric perception, such coordinate transformation will go beyond the current view of the traditional temporal lobe memory system (Wang et al., 2020). However, it remains completely unknown whether such universal functions exist or where/how they are implemented. One working hypothesis is that the thalamus performs a multiplexor function for multiple information streams, in which various thalamic nuclei relay and process multisensory input to every cortical region (including sensory cortices and frontal cortex) through reciprocal projections. Bursting thalamic neurons, in coordination with neural oscillations (e.g., theta and gamma rhythms), may be a candidate for the role of multiplexing (Akam and Kullmann, 2014; Mease et al., 2017). This hypothesis may be partially tested by disrupting specific pathways in the sensory system or inactivating specific sensory thalamic nuclei. If the first two or three predictions are correct, new experimental designs can be further considered for freely foraging rodents.

In parallel with these four key predictions, we also envision that the posterior cortex plays a subserving role in allocentric sensory perception. The parietal cortex has been known for its bridging role between perception, action, and cognition (Gottlieb, 2007), and may contribute to computing egocentric-to-allocentric transformation (Rolls, 2020). Because of the unique location of the posterior parietal cortex (PPC) as well as its projections to sensory cortices, entorhinal cortex and the frontal cortex (Wilber et al., 2015), and given the reported evidence of spatially-modulated PPC cell types in animal studies (Whitlock, 2014, 2017; Esteves et al., 2021) (Figure 3), it is not unreasonable to hypothesize that the grid-like responses of S1 and V2 neurons may be regulated by the information from and to the posterior cortex. This causal link may be experimentally tested by optogenetic PPC inactivation in rodent experiments.

Finally, we should remark that prediction in any scientific field is difficult and often proven wrong, and it will be likely no exception for our predictions. However, even if all of our predictions are wrong, this thinking process may well still be useful to provide future experiment guidelines and provoke new research questions in neuroscience. Alternatively, it would be good to design experiments to prove the opposite (i.e., test the negative).

Thus far, we have focused on our discussion and prediction in the cortex, but these criteria may also apply to subcortical areas. The search for grid codes may further go beyond mammalian brains, such as in birds (Sherry et al., 2017; Payne et al., 2021). Whether grid-like computation is a universal code for localization of generalized concepts may present itself as one of the fundamental questions in systems neuroscience. Until the complete answer is revealed, the search will continue.

## Data Availability Statement

The original contributions presented in the study are included in the article, further inquiries can be directed to the corresponding authors.

## Author Contributions

ZSC and S-JZ conceived this research, participated in the editing, and revisions of the manuscript. XL performed the rat experiments and electrophysiological recordings. XZ performed computational modeling and simulations. ZSC wrote the paper. All authors contributed to the article and approved the submitted version.

## Funding

S-JZ was supported by the National Natural Science Foundation of China (Grant Number 31872775). ZSC was partly supported by the grant from the US National Institute of Mental Health (R01-MH118928).

## Conflict of Interest

The authors declare that the research was conducted in the absence of any commercial or financial relationships that could be construed as a potential conflict of interest.

## Publisher's Note

All claims expressed in this article are solely those of the authors and do not necessarily represent those of their affiliated organizations, or those of the publisher, the editors and the reviewers. Any product that may be evaluated in this article, or claim that may be made by its manufacturer, is not guaranteed or endorsed by the publisher.
